# HomeRun Vector Assembly System: A Flexible and Standardized Cloning System for Assembly of Multi-Modular DNA Constructs

**DOI:** 10.1371/journal.pone.0100948

**Published:** 2014-06-24

**Authors:** Ming V. Li, Dip Shukla, Brian H. Rhodes, Anjali Lall, Jingmin Shu, Branden S. Moriarity, David A. Largaespada

**Affiliations:** 1 Division of Endocrinology and Diabetes, Department of Medicine, University of Minnesota, Minneapolis, Minnesota, United States of America; 2 Department of Genetics, Cell Biology and Development, University of Minnesota, Minneapolis, Minnesota, United States of America; 3 Department of Agronomy and Plant Genetics, University of Minnesota, St. Paul, Minnesota, United States of America; 4 Center for Genome Engineering and Institute of Human Genetics, University of Minnesota, Minneapolis, Minnesota, United States of America; 5 Masonic Cancer Center, University of Minnesota, Minneapolis, Minnesota, United States of America; University of Houston, United States of America

## Abstract

Advances in molecular and synthetic biology call for efficient assembly of multi-modular DNA constructs. We hereby present a novel modular cloning method that obviates the need for restriction endonucleases and significantly improves the efficiency in the design and construction of complex DNA molecules by standardizing all DNA elements and cloning reactions. Our system, named HomeRun Vector Assembly System (HVAS), employs a three-tiered vector series that utilizes both multisite gateway cloning and homing endonucleases, with the former building individual functional modules and the latter linking modules into the final construct. As a proof-of-principle, we first built a two-module construct that supported doxycycline-induced expression of green fluorescent protein (GFP). Further, with a three-module construct we showed quantitatively that there was minimal promoter leakage between neighbouring modules. Finally, we developed a method, *in vitro* Cre recombinase-mediated cassette exchange (RMCE) cloning, to regenerate a gateway destination vector from a previous multisite gateway cloning reaction, allowing access to existing DNA element libraries in conventional gateway entry clones, and simple creation of constructs ready for *in vivo* RMCE. We believe these methods constitute a useful addition to the standard molecular cloning techniques that could potentially support industrial scale synthesis of DNA constructs.

## Introduction

Modern molecular biology owes much to the invention of molecular cloning which creates recombinant DNA molecules, and allows individual DNA elements to be studied in detail. The knowledge accumulated since its invention half a century ago has not only helped elucidate the molecular basis of life, but also directly gave rise to a new branch of science, i.e., synthetic biology, the engineering arm of molecular biology. With a repertoire of DNA elements of diverse functions, they can be recombined to form functional modules, pathways, and genetic circuits to serve a predesigned purpose for the benefit of human kind [1].

Despite development of many new technologies, restriction endonuclease based methods remain the cornerstone of molecular cloning. The commonly used restriction endonucleases recognize palindromic sequences around 4–8 bp in length, therefore their chance of random occurrences in a large and complex DNA construct is prohibitively high, around 1 in every 4 kb for a typical restriction endonuclease that recognizes 6 bp. For each cloning step they must be individually selected based on restriction analysis of both the insert and vector. The steps involved also need to be carefully choreographed so that the restriction enzymes used in later steps do not cut fragments inserted earlier. Once built, the construct is often nearly impossible to modify. For these reasons, despite what was implied by “engineering” in its name, genetic engineering is still an art that requires advanced craftsmanship and thoughtful efforts, and, as such, is not amenable for automation or high-throughput production.

Efforts have been made to apply the principles of modern engineering to the field of synthetic biology, namely, by standardizing the DNA parts and their assembly process, such as BioBricks [2], Golden Gate [3], and GoldenBraid systems [4]. These methods heavily rely on restriction endonucleases, therefore require that the DNA elements do not contain any of the restriction sites to be used, a rather severe restriction. Methods based on fusion of PCR products, such as Gibson Assembly [5], Sequence and Ligase Independent Cloning (SLIC) [6], Circular Polymerase Extension Cloning (CPEC) [7], and Seamless Ligation Cloning Extract (SLiCE) [8] have been described and are highly efficacious. An effort to standardize these approaches using computer-designed bridging linkers was recently reported, known as Modular Overlap-Directed Assembly with Linkers (MODAL) [9]. However, PCR in itself is difficult to standardize, as the reaction conditions, primer design, and characteristics of the templates such as length, complexity and GC content all need to be taken into consideration for each individual amplicon, some of which could be extremely challenging to amplify. Mutations generated with PCR are also a significant concern, especially for large and complex DNA constructs. Their products, once finished, are also not amenable to revisions, as are often required.

In this study, we endeavour to create an alternative standard for assembly of multi-modular DNA constructs, which provides unrestricted accommodations for any DNA elements of interest, maintains their fidelity during the assembly processes, and also maximizes flexibility by making the parts, modules and pathways easily interchangeable for possible revisions.

One candidate approach could be cloning with homing endonucleases, which recognize specific 12–40 bp DNA sequences with extremely rare random occurrence (none or a few in a mammalian-sized genome) [10]. These sequences are also non-palindromic, therefore allowing directional cloning after a single digestion [11]. However, so far only 4 homing endonucleases are commercially available, though many more have been studied and may be commercialized soon. Their reaction conditions are quite variable, therefore only sequential addition of one module at a time is allowed, a laborious and prolonged process if every single element is to be assembled in this fashion. It is therefore not practical for them to substitute for the restriction endonucleases.

Recombinase based cloning strategies have become a convenient alternative for restriction/ligation cloning. The most popular example, gateway cloning, takes advantage of λ integrase which mediates recombination between specific attL and attR sequences [12], [13]. Remarkably, by introducing mutations to these recognition sites, multiple variants of attL and attR pair have been created with similar recombination efficacy and specificity, thus allowing multisite gateway cloning to directionally link up to 4 DNA elements in a single reaction [14], [15]. However, multisite gateway cloning by itself could not replace restriction/ligation cloning either, as the number of the elements allowed is limited to 4 or less. Site-specific recombination using Cre recombinase retrofitting has also recently been described to assemble multigene vectors [16], but is also similarly limited in capacity by the number of available loxp variants.

A combination of these methods, however, could potentially satisfy most of our previously stated goals, i.e., specificity, efficacy, fidelity, flexibility and easy standardization. For example, multisite gateway cloning has been successfully combined with Gibson Assembly, allowing rapid modular construction of multigene circuits with up to 11 transcription units [17]. Even though highly efficient, this method still leaves some extra flexibility to be desired, since the final construct could not be further revised. To further improve the flexibility of modular cloning, we present a new cloning scheme, termed HomeRun Vector Assembly System (HVAS), to take advantage of both multisite gateway cloning and homing endonucleases, with the former building modules from DNA elements, while the latter assembling modules into a final construct. We show here that, starting from a DNA element library in the form of gateway entry clones, no restriction endonucleases or PCR are required for building a functional multi-modular DNA construct, and as such the entire cloning process could be standardized. We were able to show that large multi-modular constructs built with the HVAS method in *piggyBac* backbone proved to serve their pre-designed functions after being stably transfected into mammalian cells, and minimal cross-modular leakage or noise was observed in quantitative reporter assays. We also developed a new cloning technique, termed *in vitro* Cre RMCE cloning, used here to (but not limited to) regenerate a destination vector after a previous multisite gateway cloning reaction. This allows the vast amount of pre-existing DNA elements in conventional gateway entry clones, such as full-length cDNA libraries, to be available for HVAS cloning. Overall, we believe these two innovations could greatly simplify the process for assembly of multi-modular constructs, which could now be standardized and streamlined, minimizing human efforts and error, and making industrial-scale automation and production possible.

## Materials and Methods

### Plasmid Construction

All restriction endonucleases, homing endonucleases, calf intestinal alkaline phosphatase (CIP), T4 DNA ligase, and T4 polynucleotide kinase were purchased from New England Biolabs (Ipswich, MA). Conventional and multisite gateway cloning were performed with LR clonase II and LR clonase II plus, respectively, following manufacturer's instruction (Life Technologies, Grand Island, NY). Oligonucleotides were synthesized by Integrated DNA Technologies (Coralville, Iowa). Plasmid Qiaprep miniprep kit and Qiaquick gel purification kit were purchased from Qiagen (Hilden, Germany). The pENTR vectors for building the DNA element library, including pENTR-L1/R5, pENTR-L1/L4, pENTR-L5/L4, pENTR-R4/R3, pENTR-L3/L2 and pENTR-L5/L2, were created by modification of pENTR221 (Life Technologies). Briefly, the fragment spanning attL1 and attL2 sites was first replaced with a multiple cloning site (MCS); then indicated attL or attR variants, synthesized as adaptors, were sequentially added to the MCS. For 4-element assembly, the entry clones to use include L1/R5, L5/L4, R4/R3, and L3/L2; 3-element, L1/L4, R4/R3, and L3/L2; 2-element, L1/R5 and L5/L2 [15]. For creation of pBASE destination vectors, an adaptor composed of one pair of homing endonuclease recognition sites were cloned into MCS of pBluescript II KS (+) vector (Agilent Technologies, Santa Clara, CA); subsequently, a destination cassette released from pPB-T11-DEST (B.S.M. and D.A.L, unpublished data), a gateway destination vector in a *piggyBac* transposon backbone allowing tight doxycycline-inducible expression of genes-of-interest [18], was then inserted between the homing endonuclease sites. The pairs of homing endonuclease sites used for each pBASE vector are as follows: pBASE1, I-SceI; pBASE2, I-CeuI; pBASE3, PI-SceI; and pBASE4, PI-PspI. ploxPN-DEST, the destination cassette donor for *in vitro* Cre RMCE, was created in a similar fashion. Briefly, an adaptor with following components, AscI – loxP – MCS – loxN – AscI [19], was synthesized and cloned into pBluescript II KS (+) to arrive at ploxPN-MCS; then the destination cassette was cloned into the MCS. Similarly, the donor plasmid for puromycin resistance gene (puroR) for *in vivo* cassette exchange experiments was created by cloning the coding sequence of puroR into the MCS of ploxPN-MCS. The pHR assembly vector was modified from pPB-T11-DEST as well. Briefly, the fragment between the *piggyBac* ITRs was replaced with an adaptor that contains four homing endonuclease recognition sites, including I-SceI, I-CeuI, PI-SceI, and PI-PspI. For transferring modules in pBASE shuttle vectors to pHR backbone, both plasmids were digested with the selected homing endonuclease according to the manufacturer's instruction, followed by gel purification of both the insert and vector. The vector was treated with CIP (NEB) to prevent self-ligation then column purified with Qiaquick PCR purification kit (Qiagen), before ligation with the insert with T4 DNA ligase according to standard molecular cloning protocol.

### Cell Culture and Transfection

HCT116 cells were obtained from ATCC (Manassas, VA), and cultured in DMEM high glucose media supplemented with 10% FBS plus 1% penicillin/streptomycin, at 37°C with 5% CO2. Transfection was carried out with lipofectamine 2000 reagent per manufacturer's instruction (Life Technologies). Briefly, for stable transfection of *piggyBac* transposon constructs, HCT116 cells were seeded in 6-well plates the day prior to reach 80–90% confluence at the time of transfection; for each well, 1 µg of the transposon construct and 1 µg of a plasmid expressing super *piggyBac* transposase (System Biosciences, Mountain View, CA) were mixed with 6 µl of lipofectamine 2000 reagents in 300 µl of serum free opti-MEM media, which were then added to cells for incubation over night. For *in vivo* cassette exchange, 1 µg of purified floxed PuroR cDNA and 1 µg of pRN-Cre, an expression plasmid for Cre recombinase, were used for each transfection. Selection with appropriate antibiotics occurred 24 hours after transfection. G418 was used at 1 mg/ml, and puromycin 1 µg/ml.

### Fluorescent Microscopy and Imaging

Live cell imaging was performed with a Leica DM IL inverted microscope (Leica Microsystems, Buffalo Grove, IL). Images were acquired with Leica Application Suite v3.8 (Leica Microsystems) following the manufacturer's instructions.

### *In Vitro* Cre RMCE Cloning

Recombinant Cre recombinase was purchased from NEB. The destination cassette was released from donor plasmid ploxPN-DEST by digestion with AscI, followed by electrophoresis and gel-purification. The purified donor fragment and the recipient plasmid, which also contained the loxP/loxN pair in the same orientation, were mixed (100 ng each) with 1 unit of Cre with provided buffer in a 20 µl reaction, which was allowed to proceed at 37°C for 30 minutes. The reaction was then transformed into OneShot ccdB Survival 2 T1R competent *E. coli* (Life Technologies), which were then selected with both chloramphenicol and another antibiotic appropriate for the recipient plasmid.

### Luciferase Assay

Activities of *firefly* and *renilla* luciferases were determined by dual luciferase assay kit (Promega, Madison, WI) according to the manufacturer's instruction. The readings were normalized to the protein content of each sample as determined by Bradford assay (Sigma, St Louis, MO). Both luciferase and Bradford assays were carried out on a Synergy 2 microplate reader (Biotek, Winooski, VT).

### Statistical Analysis

Unpaired Student's *t*-tests were used for data comparison where appropriate, with p<0.05 suggestive of statistical significance, with Windows Excel for Mac 2011 (Microsoft, Seattle, WA).

## Results

### HomeRun vector assembly system: a three-tiered vector system for modular assembly of DNA molecules

**Figure 1:**
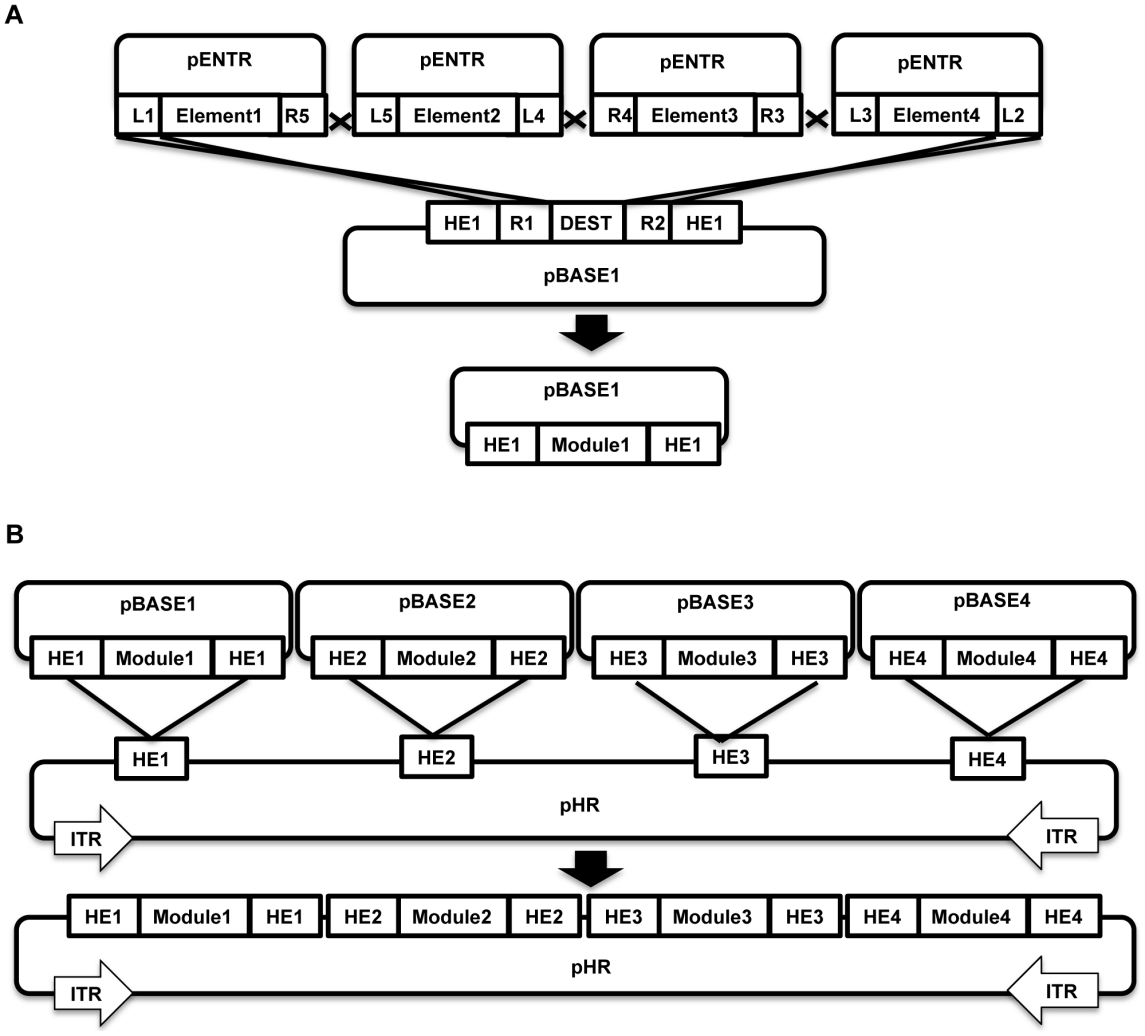
Schematic illustration of the HVAS vector system and its assembly process. 1A, Assembly of a functional module in pBASE vectors from elements in pENTR vectors; 1B, Assembly of multi-modular construct in pHR assembly vectors from modules in pBASE shuttle vectors. Up to 4 elements in pENTR vectors can be assembled on each pBASE shuttle vector, and up to 4 modules in the pBASE shuttle vectors can sequentially dock onto the pHR assembly vector. Abbreviations: L1, attL1; R5, attR5; L5, attL5, L4, attL4; R4, attR4; R3, attR3; L3, attL3; L2, attL2; R2, attR2; R1, attR1; HE, homing endonuclease; DEST, destination cassette; HE1, I-SceI; HE2, I-CeuI; HE3, PI-SceI; HE4, PI-PspI; ITR, inverted terminal repeat.

We reasoned that multisite gateway cloning could be an ideal tool for building functional modules, since most of them are transcription units composed of three to four elements, including promoter, cDNA, polyA signal, and sometimes regulatory sequences such as insulators and silencers. Given the high efficacy of the reaction, multisite gateway cloning could build a functional module in a single reaction. Homing endonucleases on the other hand could be a great tool to assemble the modules together in lieu of restriction endonucleases. The modules would be freely interchangeable in the final construct, allowing the latter to be easily modified. Most importantly, without reliance on restriction enzymes or PCR, the entire cloning process can potentially be standardized and streamlined on an industrial scale. To realize this rationale, we created a three-tiered vector system termed HomeRun Vector Assembly System, in honor of the Homing Endonucleases and Recombinases used in this design (Fig. 1).

At the parts level, we made use of the pENTR vectors with attL and attR variants for multisite gateway cloning as the standard format for depositing all DNA elements. Each attL variant specifically recombines with its synonymous attR counterparts, and up to 4 of these elements can be linked in a single clonase reaction (Fig. 1A). The DNA elements to be assembled could be cloned into these entry clones either as PCR products with BP clonase [15], or by conventional restriction/ligation cloning, using a MCS placed between the att variants in the pENTR vectors. This flexibility in methods of deposition of DNA elements ensures that most DNA elements-of-interest can be accommodated by the HVAS system, without any restriction imposed by previous efforts, such as lack of certain restriction sites (for BioBrick or Golden Gate), or being easily amplifiable by PCR (for PCR-fusion based methods).

With the choices of 6 pENTR vectors, it is important to have a certain rule for depositing diverse DNA elements, even though for maximal flexibility, the same element can be deposited in all 6 pENTR vectors so that it could be readily used in any possible combinations for 2, 3, or 4-element assembly. As most of the modules required in our practice are transcription units composed of promoter, cDNA and polyA signal, we typically place promoters in pENTR-L1/L4 (for 3-element modules) and pENTR-L5/L4 (for 4-element modules), cDNAs in pENTR-R4/R3 and polyA signals in pENTR-L3/L2, which can then be directly used to build a transcription unit. Additional regulatory sequences such as an insulator are deposited in pENTR-L1/R5 for 4-element modules. Following these simple rules, we have built a small yet expanding library of useful elements, with those used in this study listed in Table 1. In the future, we aspire to build an all-inclusive library of relevant DNA elements with the collective efforts from the entire molecular and synthetic biology community, as a complementary alternative to a similar one envisioned by BioBricks Foundation [2]. Efforts in this direction have already been underway from several different groups, resulting in a few collections of multisite gateway entry clones and destination vectors made openly available to the research community [20], [21]. This library could serve as a starting point upon which no further restriction endonuclease or PCR reactions will be required for building most of the desired DNA constructs, regardless of their size and complexity, with all involved reactions standardized.

**Table 1:** Part of the DNA element library used in this study.

Element	Entry vector	Annotation
TRE	pENTR-L1/L4	Tetracycline response element [18]
GFP	pENTR-R4/R3	green fluorescent protein (gb|KF528987.1|, nucleotides 2013–2372 *)
SV40PA	pENTR-L3/L2	SV40 polyA signal (gb|KF528988.1|, 1027–1228 *)
pEF1a	pENTR-L1/L4	EF1α promoter (gb|EU424173.1|, 3695–4862 *)
rtTA-IRES-puroR	pENTR-R4/R3	Reverse tetracycline-transactivator (gb|DQ349228.1|, 1368–2065 *)– internal ribosome entry site (gb|KC262216.1|, 7596–8181 *) – puromycin resistance (emb|Z75185.1|, 1694–2296 *)
BGHPA	pENTR-L3/L2	Bovine Growth Hormone PolyA Signal (gb|JQ624676.1|, 791–1015 *)
fLuc	pENTR-R4/R3	*Firefly* luciferase (gb|JN542721.1|, 280–1929 *)
pCMV	pENTR-L1/L4	CMV promoter (gb|KF366485.1|, 2425–3012 *)
rLuc	pENTR-R4/R3	*Renilla* luciferase (gb|AF025846.2|, 1034–1969 *)
pCAG	pENTR-L1/L4	CAG promoter (gb|JQ627827.1|, 3413–5035 *)
loxPN	pENTR-R4/R3	LoxP and LoxN in tandem [19]

* Genbank accession number and nucleotide location of indicated elements.

At the next level of complexity, i.e., functional modules, we have developed four vectors called pBASE1, 2, 3 and 4 respectively, which serve as not only the platforms on which the DNA elements could be assembled into functional modules (Fig. 1A), but also shuttle vectors that could dock these modules into the assembly frame for the final construct by homing endonucleases (Fig. 1B). They are named pBASE for HomeRun's reference to a baseball game, in particular with the four vectors mimicking the four bases. Essentially, these vectors are gateway destination vectors with the destination cassette flanked by a pair of synonymous homing endonuclease sites (Fig. 1A). Similarly, a module library could be built that could be readily used for DNA constructs of higher-level complexity.

Finally, at the level of genetic circuits, we have created pHR vector to serve as the assembly frame onto which each functional module could be docked (Fig. 1B). The docking ports in pHR are essentially a tandem array of recognition sites for the 4 homing endonucleases currently commercially available, i.e., I-SceI, I-CeuI, PI-SceI, and PI-PspI. Functional modules could be individually released from pBASE vectors and inserted into their corresponding ports in pHR. For this study, the prototype pHR vector is created in a *piggyBac* transposon that could facilitate the stable genomic integration of the construct with a cargo size up to >100 kb [22]. The *piggyBac* backbone is particularly suitable for mammalian cell models with the allowed cargo size, but other backbones such as phage, virus, bacterial artificial chromosomes (BACs), or even a prokaryotic genome, are equally appropriate. At this stage, up to 4 modules can be added sequentially to the final construct with homing endonuclease/ligase reactions based on the HVAS system, but could be expanded to up to 16 by incorporating additional technologies developed in our lab (Figs. S1 and S2, see discussion for details). In the long run, with more homing endonucleases being made available, and also the possibility to use designer endonucleases such as Zinc Finger Nucleases (ZFNs) [23], Transcription Activator-Like Effector Nucleases (TALENs) [24], and CRISPR [25], [26], the level of complexity that could be supported is essentially unlimited.

### Creation of a functional doxycycline-inducible expression system with the HVAS approach

**Figure 2:**
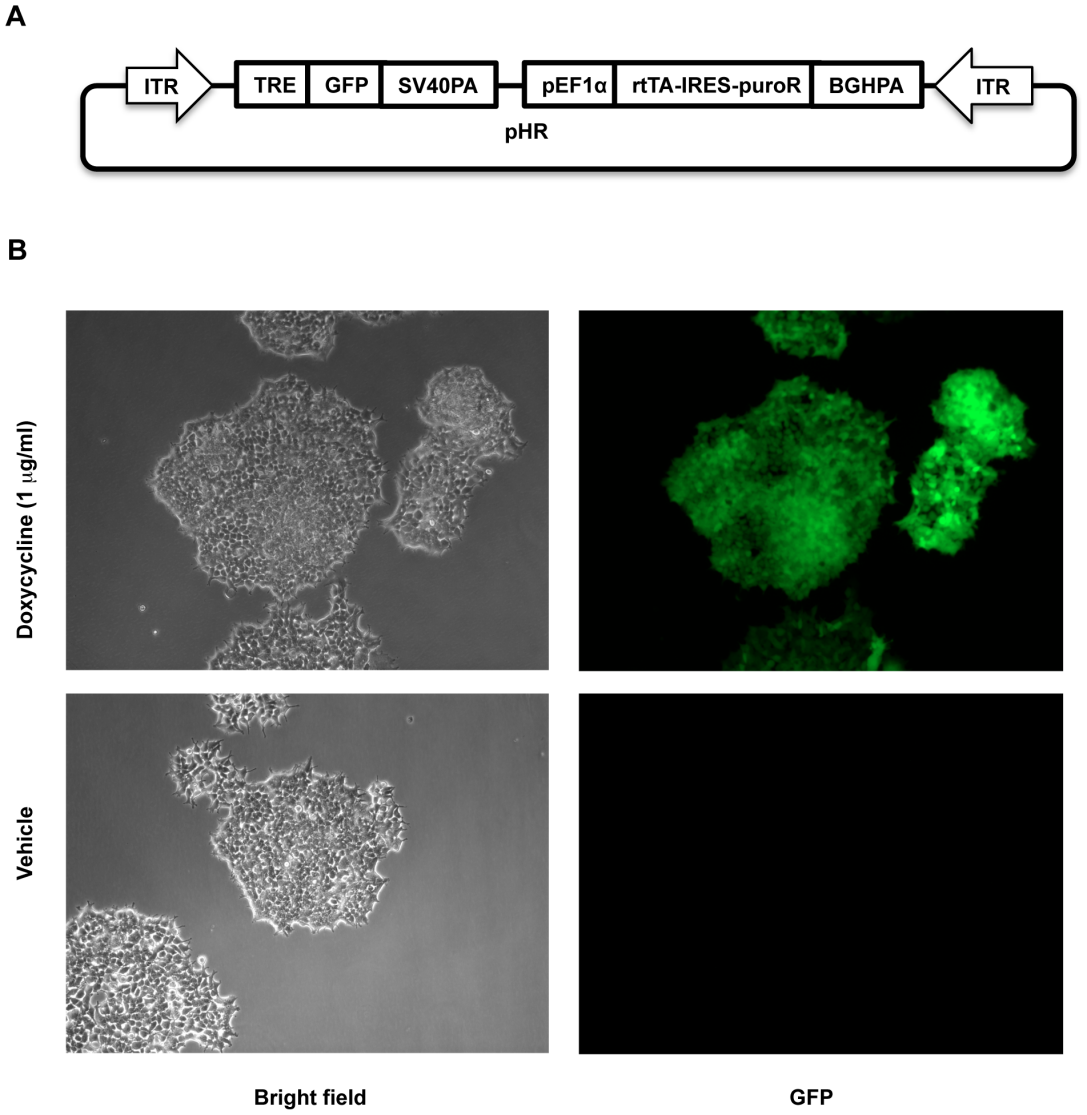
Building of a functional two-module construct with HVAS which supported doxycycline-induced GFP expression. 2A, schematic illustration of the construct. The abbreviations of depicted elements were annotated in Table 1. 2B, above construct was stably transfected into HCT116 cells, and selected with 1 µg/ml puromycin. Surviving cells were treated with or without 1 µg/ml doxycycline over night before image acquisition with bright field and fluorescent microscopy.

As a proof-of-principle for the HVAS method, we made a two-module construct that supports doxycycline inducible expression of a GFP reporter gene. The first module was composed of three elements, i.e., tetracycline-inducible promoter, cDNA for GFP, and a SV40 polyA signal in that order, while the second module contained EF1α promoter, cDNA for rtTA-IRES-puroR and BGH polyA signal. The first module supports expression of GFP in the presence of rtTA and doxycycline while the second one provides rtTA and puromycin resistance for stable selection. The two modules were then sequentially assembled into the pHR backbone to build one of the simplest yet functional binary systems (Fig. 2A). Of note, we observed high efficiency for both multisite gateway cloning reactions, similar to those previously reported [15], [21], and homing endonucleases/ligation cloning, with all 4 colonies isolated in each cloning step being correct in the majority of cases.

After the final construct was stably transfected into HCT116 cells facilitated by *piggyBac* transposase, we were able to show that the cells did not express detectable GFP without doxycycline using fluorescence microscopy, but in the presence of doxycycline, strong and uniform expression of GFP could be observed (Fig. 2B). The above results clearly validated the concept of HVAS cloning strategy, suggesting that it could be generally applicable in other settings.

### Minimal cross-modular noise in a multi-modular construct

The use of HVAS in synthetic biology is to build functional multi-modular genetic circuits. Often these circuits are required to process and output multiple signals. With the modular organization of transcription units, the expression of one gene is likely subject to unintended influence from the promoter or regulatory sequence of a nearby transcription unit, contributing to the noise of the system. It is well known that promoters within such a multi-modular construct are subject to promoter interference, i.e., significant diminishment of transcriptional activity, which could be alleviated by separating neighboring transcription units by chicken β-globin insulator (cHS4) [16], [27]. The opposite, i.e., enhanced expression of a gene from leakiness of a nearby promoter, however, is less well studied in such settings. This is likely due to the difficulty in deciding the portion of the promoter activity derived from the leakiness of a nearby module. One possible solution, we reasoned, could be to use a drug-inducible promoter as a source of interference, since its effects on a separate downstream module could be quantitatively measured by the latter's response to the inducing agent. We therefore decided to quantitatively investigate the extent of cross-module promoter leakiness in a multi-modular construct built by HVAS system with this approach.

**Figure 3:**
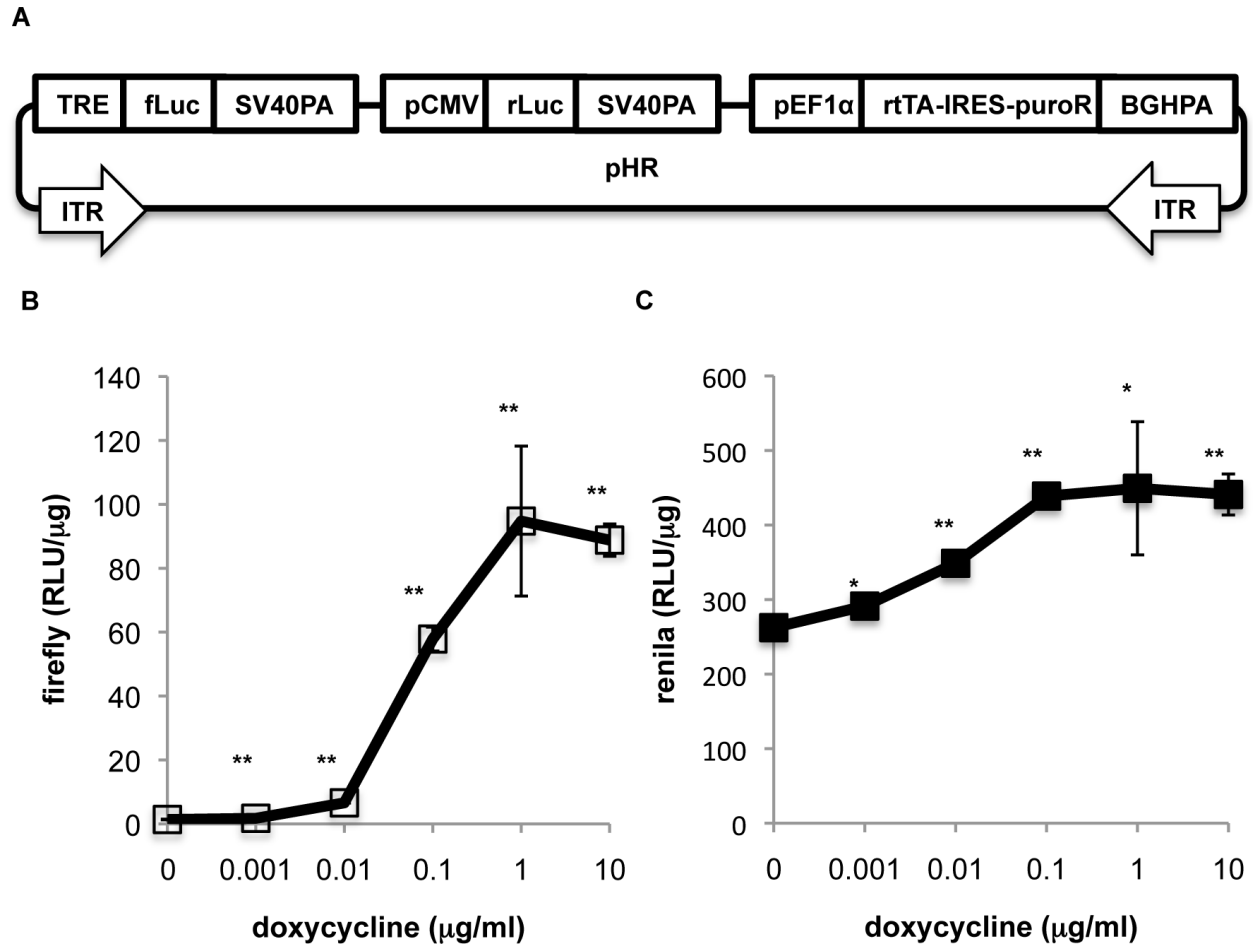
Minimal cross-modular promoter leakiness was observed with dual luciferase assay. 3A, Schematic illustration of the three-module construct. The abbreviations of depicted elements were annotated in Table 1. 3B, and 3C, response of *firefly* (3B) and *renilla* (3C) luciferase activities to increasing concentrations of doxycycline; the above construct was stably transfected into HCT116 cells and selected with 1 µg/ml puromycin. Surviving cells were seeded in 24-well plates the day prior to treatment to reach around 80% confluence at the time of treatment; vehicle or doxycycline of indicated concentrations were added for incubation for 24 hours at 37°C before dual luciferase assay. Each treatment was triplicated. Results were represented as relative light units normalized to protein content (RLU/µg). Comparisons were made between results in samples treated with indicated doxycycline concentration and those treated with vehicle only (doxycycline 0 µg/ml). *, p<0.05; **, p<0.01.

We started by building a three-modular construct in pHR backbone with HVAS approach (Fig. 3A). From 5′ to 3′, module 1 is composed of tetracycline-inducible promoter, coding sequence of *firefly* luciferase and SV40 polyA signal; module 2, CMV promoter, *renilla* luciferase cDNA, and SV40 polyA signal; and module 3, EF1α promoter, rtTA-IRES-PuroR, and BGH polyA signal. We have intentionally placed the second reporter (CMV-*renilla* luciferase) downstream to the first, so that it is prone to the interfering effect of the upstream external promoter, i.e., the TRE promoter. The construct was stably transfected into HCT116 cells using *piggyBac* transposase. The response of *renilla* luciferase activity to doxycycline provided a quantitative surrogate measurement for cross-modular influence from the TRE promoter upstream. After these cells were treated with increasing concentration of doxycycline, a 70-fold increase in the *firefly* luciferase activity was observed as expected (Fig. 3B); at the next module, there was also a small but statistically significant 1.7-fold increase in the *renilla* luciferase activity. In this particular experiment, the high baseline level of *renilla* luciferase activity caused by the CMV promoter might partly explain the minimal leakiness. However, this experiment did prove that cross-module promoter leakiness exists, but at least in this setting was not strong. If stronger leakiness is experienced, it could be envisioned that regulatory sequences such as insulators could be employed to improve the signal to noise ratio. Currently, it is safe to conclude that multi-modular constructs built with HVAS are suitable for complex genetic circuits with manageable levels of cross-modular noise.

### Regenerating gateway destination vector with *in vitro* Cre RMCE

**Figure 4:**
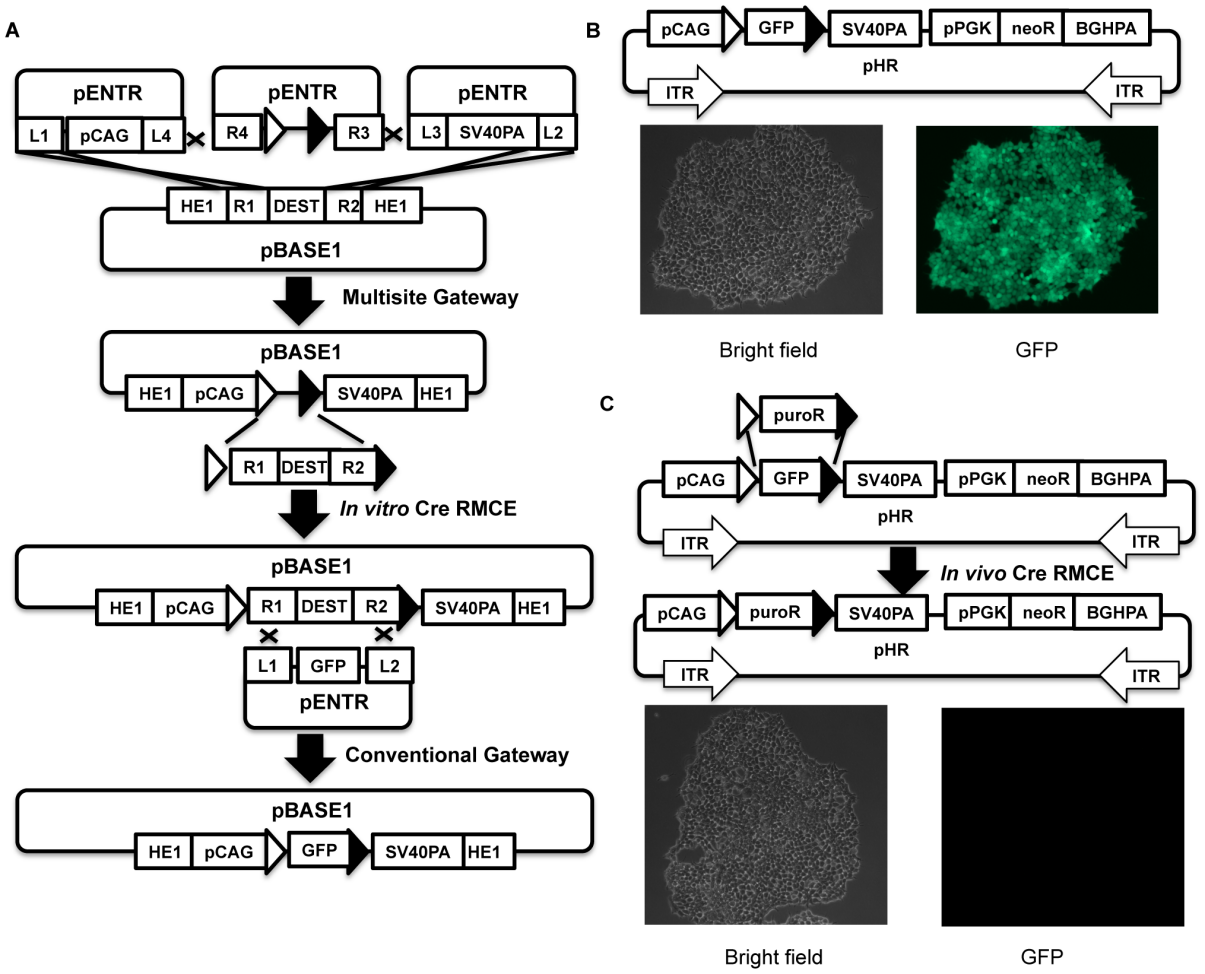
*In vitro* Cre RMCE cloning and its application in HVAS. 4A, schematic illustration of regeneration of a destination vector with *in vitro* Cre RMCE cloning. The abbreviations of depicted elements were annotated in Table 1. 4B, fluorescence microscopy for HCT116 cells stably transfected with a 2-module construct built with HVAS and *in vitro* Cre RMCE cloning as illustrated. Open triangle, canonical loxP; solid triangle, loxN variant. 4C, *in vivo* RMCE, the cells shown in 4B were transfected with floxed puroR cDNA along with pRN-Cre, selected with both puromycin (1 µg/ml) and G418 (1 mg/ml). Fluorescence and bright field imaging of representative colonies were shown.

Multisite gateway cloning is a useful expansion to the conventional gateway system. The latter is carried out by the same attL/R recombination between a single entry clone with attL1 and attL2, and a destination vector with attR1 and attR2. It is apparent that both multisite and conventional gateway methods share the same recombination sites attR1 and attR2, therefore are mutually exclusive (Fig. 4A). As a result, an entry clone for conventional gateway cloning is not usable for our HVAS scheme. This is unfortunate since conventional gateway entry clones with attL1 and attL2 have become the standard format for many full-length cDNA or ORF libraries [28], [29], along with many other important DNA elements.

To make these resources readily available for HVAS cloning, we have developed a new cloning technique, i.e., *in vitro* Cre RMCE cloning (Fig. 4A). When combined with multisite gateway cloning, this technology allowed us to regenerate a gateway destination vector in a two-step cloning process, which in turn became an appropriate recipient for conventional entry clones (Fig. 4A).

Recombinase-mediated cassette exchange (RMCE) based on either Cre/loxp or flp/frt systems has been used extensively *in vivo* for gene targeting in eukaryotic and prokaryotic genomes. This allows targeting transgenes precisely to the loci flanked by the loxp or frt variants, and has become a useful tool for gene therapy and genomic research [30], [31]. Similar strategy has also been employed in gateway cloning, i.e., site-specific recombination *in vitro* with λ integrase, with great success [13]. Attempts to use Cre *in vitro* for RMCE cloning, however, were not successful for unclear reasons [13], except that the efficiency for *in vitro* Cre RMCE was recently reported to be as low as 5% [32]. So far, the listed uses for recombinant Cre enzyme does not include RMCE, but is limited to excision, inversion or fusion of DNA from the manufacturer's description (https://www.neb.com/products/M0298-Cre-Recombinase).

For our purpose, we realized that *in vitro* Cre RMCE cloning could be a useful bridging step to regenerate a gateway destination vector from the product of a previous multisite gateway cloning, with a scheme depicted in Fig. 4A. We reasoned that even with a relative low efficiency, we might be able to make it work by applying appropriate selections. To test this possibility, we created a plasmid ploxPN-DEST as the donor for the destination cassette, which was flanked by a pair of loxp variants, i.e., loxP and loxN. The positive/negative selection markers contained in the destination cassette, i.e., chloramphenicol resistance (CmR) and ccdb toxin genes, were utilized for the tag-and-exchange maneuver in the Cre RMCE scheme too. We then created a recipient plasmid using multisite gateway cloning to build a module composed of three elements, i.e., CAG promoter, LoxP/LoxN pair, and SV40 polyA signal on pBASE1 platform. We initially attempted *in vitro* Cre RMCE between uncut donor and recipient plasmids, and found that most of derived colonies did not give rise to the desired products, likely the result of plasmid fusion instead of cassette exchange (data not shown). However, when the destination cassette was released by AscI digestion from ploxPN-DEST, and mixed with the uncut recipient plasmid in the presence of Cre, we were able to generate the desired product, a new destination vector that supported expression under the strong CAG promoter for any incoming genes-of-interest, such as GFP as illustrated in Fig. 4A. The reaction appeared highly specific; in a majority of cases, we were able to get 4 correct colonies out of 4 isolated, with the rest at least 3 out of 4.

The expression plasmid derived from the new destination vector is immediately ready for cloning as a module into the pHR assembly vector by homing endonucleases. As a proof-of-principle for this scheme, we created a two-module construct in pHR (Fig. 4B). The first module is made with above-described multisite gateway cloning/*in vitro* Cre RMCE maneuver as shown in Fig. 4A, which supports expression of GFP under CAG promoter. The second module expresses neomycin resistance gene (NeoR) under PGK promoter to allow for stable selection with G418. After the construct was stably transfected into HCT116 cells, all cells surviving selection with G418 showed uniform strong green fluorescence (Fig. 4B), proving that this method was indeed valid for creating functional multi-modular constructs.

It did not escape our attention that constructs built in this fashion would automatically support *in vivo* RMCE. To prove this, we co-transfected a DNA fragment that was composed of cDNA for puroR which was flanked with loxP and loxN along with a plasmid expressing Cre into the cells shown in Fig. 4B. These cells were then selected with both G418 and puromycin. With the exception of a few colonies likely resulting from unspecific chromosomal integration, a majority of surviving colonies had lost green fluorescence when examined with fluorescent microscopy (Fig. 4C), suggesting successful cassette exchange.

## Discussion

### HVAS: a standardized and flexible cloning system

We present here the HVAS cloning strategy, a new approach aiming to standardize the cloning process in the fashion of modern engineering. By organizing DNA at all levels of complexity in standardized formats, and assembling them with limited numbers of standardized reactions, the entire process can be streamlined with minimal human efforts and errors, eventually paving the way for high-throughput automatic production.

We are the first to admit that there are a few shortcomings of this system. First, there would be extensive assembly scars left in the final construct in the form of attB sequences as a result of attL/R recombination, homing endonuclease sites, and residual restriction sites from the pENTR vectors. The problem is likewise shared by many other methods such as restriction/ligation, linker-mediated PCR fusion, and site-specific recombination, but still not desirable compared to scarless assembly methods such as Golden Gate and Gibson Assembly. In our experiments this did not seem to have resulted in any problems, but their effects on larger constructs remain to be seen.

Second, currently the module capacity of the HVAS system is limited to 4 by the number of commercially available homing endonucleases; we do, however, expect this to improve with more homing endonucleases becoming available and new designer endonucleases such as ZFNs and TALENs being developed. At that point, there would not be any limit to the cargo size. In the near term, however, regenerated destination vector from combined multisite gateway cloning and *in vitro* Cre RMCE is immediately ready for another round of multisite gateway cloning, therefore the capacity for each pBASE shuttle vector could be doubled to 2, and that of the entire HVAS system to 8 (Fig. S1). Alternatively, HVAS could be combined with other high-efficacy modular assembly methods such as Cre-loxp mediated plasmid fusion (retrofitting) as utilized by the RecWay system [16]. Under this design, the number of modules that could be assembled could be at least quadrupled to 16, depending on the numbers of available loxp variants (Fig. S2A). Other site-specific recombination systems such as Dre/Rox [33] and flp/frt [30] could also be employed for further increase in capacity. These should be able to satisfy the need of most molecular and synthetic biologists. The retrofitting protocol is also amenable to standardization as all reactions are highly specific with predefined conditions. With the increased number of modules, higher levels of complexity could be supported as an extension of the hierarchy described earlier; for example, in addition to the elements and modules, pathways can be assembled with homing endonucleases, then the genetic circuits by Cre/loxp retrofitting (Fig. S2B). Importantly, libraries of functional modules and pathways can also be developed in pBASE and pHR vectors respectively, and made readily available for construction of complex genetic circuits.

At last, the stepwise addition of modules by homing endonucleases reaction is time-consuming and less efficient when more than a few modules are needed in the final construct, especially compared to the one-pot one-step solution offered by some PCR based methods such as Gibson Assembly [5], [9]. However, at the cost of efficiency, homing endonucleases offer the great advantages of flexibility, fidelity and interchangeability. Including Cre/loxp retrofitting, as described above (Fig. S2B), offers a partial solution to improve the efficiency by allowing these reactions to occur in parallel in several pHR backbones simultaneously before they are fused together. Also, establishing ready-to-use libraries of multi-modular pathways in pHR vectors can further speed up the cloning process for complex genetic circuits. Ultimately, however, designer endonucleases such as ZFNs, TALENs and CRISPR hold great promise for potential one-step assembly of multiple modules, as they can be engineered to work under the same reaction condition by employing the same endonuclease domain.

Despite all its imperfections, HVAS is likely one of the most flexible cloning systems described so far in that assembly at all levels of complexity, i.e., elements, modules, pathways, and genetic circuits, is standardized, and all the elements and the modules are freely interchangeable. It is also broader in scope compared to most previous standardization efforts in that it does not have any pre-defined limitations to the acceptable DNA parts, such as lack of certain restriction sites as entailed by BioBrick or Golden Gate, or being amplifiable by PCR for methods based on PCR fusion. There is also no concern for fidelity as no DNA polymerases are involved in any reactions. It does not require much human effort for designing cloning strategy, since it follows a natural logic of organization from simple structure to complex without requiring any individualized consideration such as selection of restriction endonucleases. Most of its shortcomings such as limited cargo size and efficiency can be solved by development of newer designer endonucleases. For these reasons, HVAS is likely one of the most suitable methods for automated and high throughput cloning at industrial scale so far.

### Quantitative measurement of cross-modular transcription interference and noise

It is unavoidable that complex genetic circuits are organized in a multi-modular structure. The human genome, in its simplest notion, is nothing but a series of massive multi-modular DNA molecules which are well demarcated in terms of structure and function. Synthetic biologists have much to learn from the organization of our own genome regarding how cross-modular interferences and noise are kept at a minimum, even though knowledge in this regard continues to accumulate, such as the role of insulator in enhancer blocking and prevention of heterochromatin spreading [34] and has been successfully applied in multi-modular constructs [16], [27]. Nonetheless, to understand how a genetic circuit works, it is essential to be able to quantitatively describe the interference and noise between different modules. In our particular example of the 3-modular system, it was apparent cross-modular noise from promoter leakiness did exist, though at a minimal level. Even though this result could hardly be readily extrapolated to other systems, this does give us confidence that the concept of multi-modular organization for genetic circuit is solid with manageable noise and interference. Much still needs to be done to better assess the noise and to optimize the module organization to best improve the signal to noise ratio.

### *In vitro* Cre RMCE as a new cloning tool

We also developed a new technology, i.e., *in vitro* Cre RMCE cloning. Compared to its more popular counterpart, i.e., gateway cloning, it is not as convenient or robust, but does provide a valid complementary alternative under certain situations, such as in this study, where it effectively regenerated a destination vector from a previous multisite gateway cloning reaction, and greatly broadened the scope and flexibility of multisite gateway cloning which can now accommodate elements in conventional gateway entry clones, and two rounds of multisite gateway cloning can be executed on a single destination vector (Fig. S1). Since the loxp variants' sequences are not altered by the reaction, the final construct can be repeatedly modified and can be readily used for *in vivo* cassette exchange once stably integrated into the genome.

In summary, we have made two related innovations. The HVAS cloning system provided a standardized and flexible cloning strategy, which we hope could serve as a platform for industrial scale sub-cloning. The *in vitro* Cre RMCE cloning also provides a useful addition to the toolkit of molecular biologists, especially in expanding the utility of multisite gateway cloning and HVAS platform, and also creating suitable constructs for *in vivo* RMCE. We believe these innovations could greatly simplify the cloning processes for most of molecular and synthetic biology applications.

## Supporting Information

Figure S1 **Schematic illustration of capacity doubling enabled by RMCE and repeated multisite gateway cloning.**
(TIF)

Figure S2 **Combined HVAS/RecWay system supports higher levels of complexity.** 2A, schematic illustration of Cre retrofitting of 3 multi-modular constructs. Open triangle, canonical loxP; solid triangle, loxN. 2B, a new hierarchal vector system based on HVAS/RecWay supports 4 levels of complexity, from elements, modules, pathways, to genetic circuits.
(TIF)
