# Supplementary figures and images for: HomeRun Vector Assembly System: A Flexible and Standardized Cloning System for Assembly of Multi-Modular DNA Constructs
Source: PLoS One. 2014 Jun 24;9(6):e100948. doi: 10.1371/journal.pone.0100948 (PMC4069157; doi:10.1371/journal.pone.0100948)

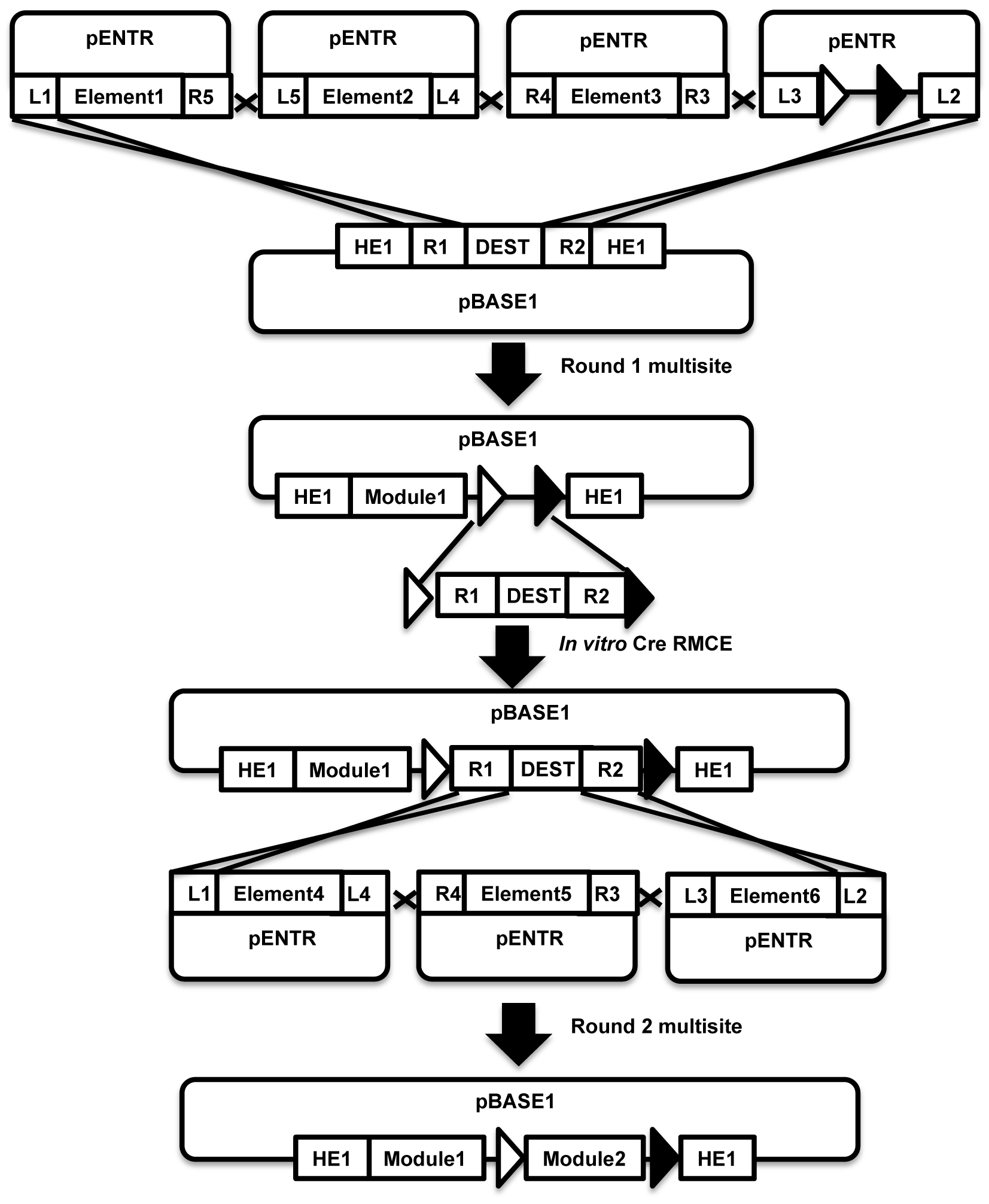

Supplement: Figure S1 — Schematic illustration of capacity doubling enabled by RMCE and repeated multisite gateway cloning. (TIF) [file pone.0100948.s001.tif]

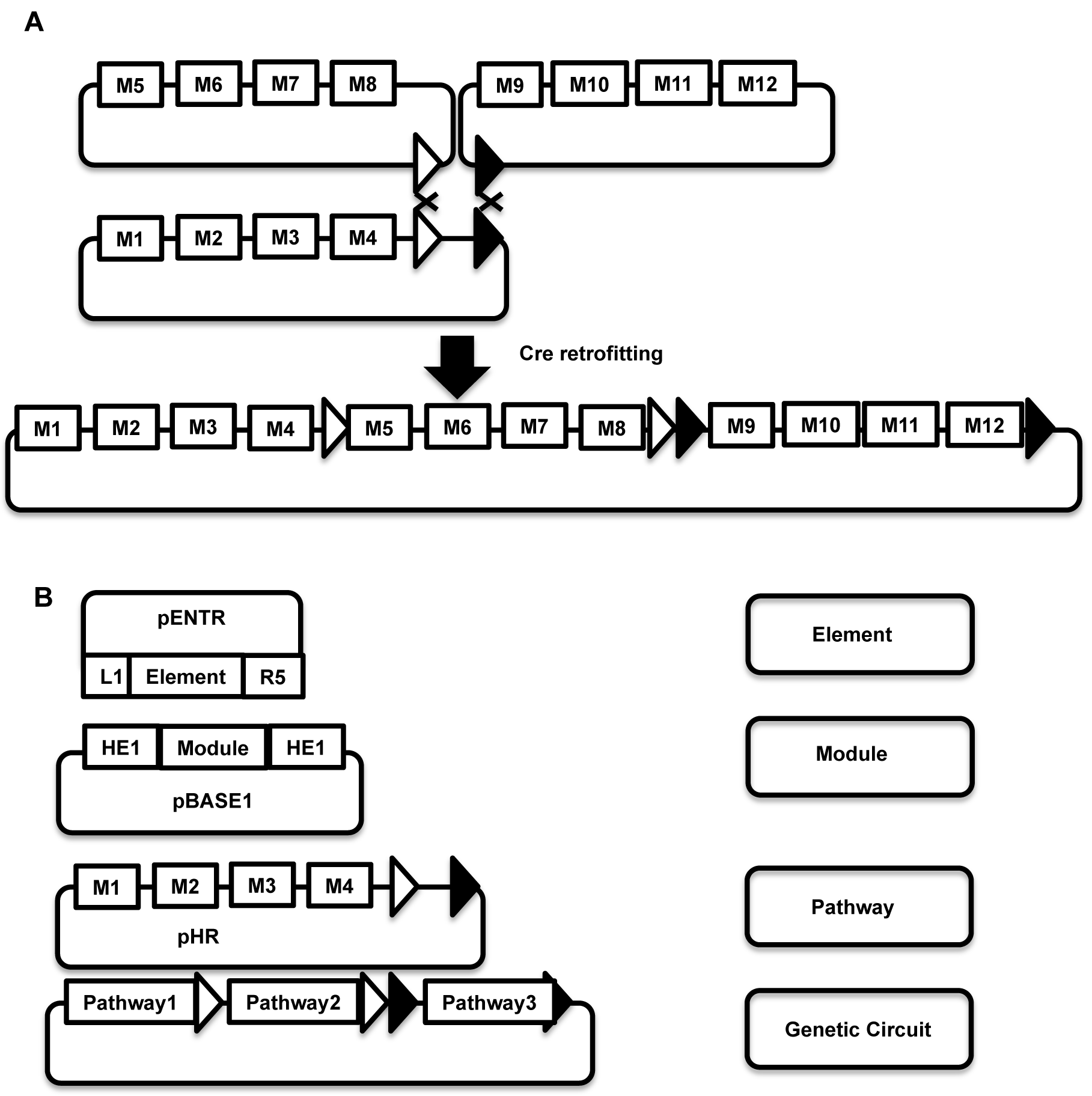

Supplement: Figure S2 — Combined HVAS/RecWay system supports higher levels of complexity. 2A, schematic illustration of Cre retrofitting of 3 multi-modular constructs. Open triangle, canonical loxP; solid triangle, loxN. 2B, a new hierarchal vector system based on HVAS/RecWay supports 4 levels of complexity, from elements, modules, pathways, to genetic circuits. (TIF) [file pone.0100948.s002.tif]
